# Mimicking the Ice Recrystallization Activity of Biological Antifreezes. When is a New Polymer “Active”?

**DOI:** 10.1002/mabi.201900082

**Published:** 2019-05-14

**Authors:** Caroline I. Biggs, Christopher Stubbs, Ben Graham, Alice E. R. Fayter, Muhammad Hasan, Matthew I. Gibson

**Affiliations:** Department of Chemistry, University of Warwick, Coventry CV4 7AL, UK; Warwick Medical School, University of Warwick, Coventry CV4 7AL, UK; Department of Chemistry, University of Warwick, Coventry CV4 7AL, UK

**Keywords:** antifreeze proteins, biomaterials, cryopreservation, ice recrystallization, polymers

## Abstract

Antifreeze proteins and ice-binding proteins have been discovered in a diverse range of extremophiles and have the ability to modulate the growth and formation of ice crystals. Considering the importance of cryoscience across transport, biomedicine, and climate science, there is significant interest in developing synthetic macromolecular mimics of antifreeze proteins, in particular to reproduce their property of ice recrystallization inhibition (IRI). This activity is a continuum rather than an “on/off” property and there may be multiple molecular mechanisms which give rise to differences in this observable property; the limiting concentrations for ice growth vary by more than a thousand between an antifreeze glycoprotein and poly(vinyl alcohol), for example. The aim of this article is to provide a concise comparison of a range of natural and synthetic materials that are known to have IRI, thus providing a guide to see if a new synthetic mimic is active or not, including emerging materials which are comparatively weak compared to antifreeze proteins, but may have technological importance. The link between activity and the mechanisms involving either ice binding or amphiphilicity is discussed and known materials assigned into classes based on this.

## 1 Introduction

Antifreeze, ice-binding and ice-nucleating macromolecules have been discovered in a diverse range of extremophile organisms from fish to insects to plants. These are produced to enable the species to survive by promoting freeze-tolerance and freeze-avoidance and have been extensively reviewed.^[1–4]^ The ability to modulate ice formation and growth has vast technological importance across fields such as aerospace,^[5]^ green energy,^[6]^ automotive, and biology, in particular cellular cryopreservation^[7–10]^; the storage of cells/tissues at subzero temperatures, which underpins many areas of modern biomedicine, clinical medicine, and basic biology. Current strategies are based on adding large volumes of (low molecular weight) cryoprotective agents such as glycerol or DMSO which enable slow freezing or vitrification.^[11–13]^ Antifreeze (glyco) proteins, (AF(G)Ps) have been found to improve cryopreservation outcomes, due to their property of ice recrystallization inhibition (IRI; discussed more below),^[14–16]^ but also potentially by stabilizing membranes.^[17,18]^ Considering this, there has been significant recent interest in developing synthetic materials to mimic AF(G)Ps. AF(G)Ps have three main properties resulting from their ice interactions; non-colligative depression of the freezing point (thermal hysteresis (TH)), dynamic ice shaping (DIS); IRI activity. The latter (IRI) is of particular interest, as it was discovered in 2003 that synthetic mimics of AFGPs can retain IRI activity,^[19]^ but not thermal hysteresis or DIS, even after major structural modifications, suggesting this property may be easier to mimic (as it may not require ice-face binding) and it has application in cryopreservation.^[20–23]^ What is clear, however, is that the synthetic mimics tend to be far less active than the natural antifreezes, yet the activity appears to be sufficient to enable beneficial cryopreservation outcomes.^[22]^ In short, it might not be that the most active IRI material is the most useful (e.g., depending on how accessible it is) and recent results suggest that IRI activity can be induced by more than one mechanism, including those which do not include ice binding.^[4,24]^

Considering the interest in introducing IRI activity into synthetic materials, the aim of this article is to provide a concise comparison of the IRI activities of a range of biological and synthetic compounds. Here we use the “splat” assay, which is a common and easily accessible technique to evaluate IRI activity, but by no means the only method. We also summarize the currently known materials with IRI, providing a snapshot of the emerging field. Based on this, we also propose a “rough guide to activity,” grouping materials from potent to very weak activity to enable researchers to decide if a new material is active or not, relative to those already published and especially for non-protein IRI active materials. It should be noted that Koop et al. have proposed a similar map based on growth rate constants, but only included highly active peptide (and PVA) ice growth inhibitors,^[25]^ whereas we intend to include examples of, and activity data for, emerging materials at all activity levels.

## 2 Measuring IRI Activity

**Figure 1:**
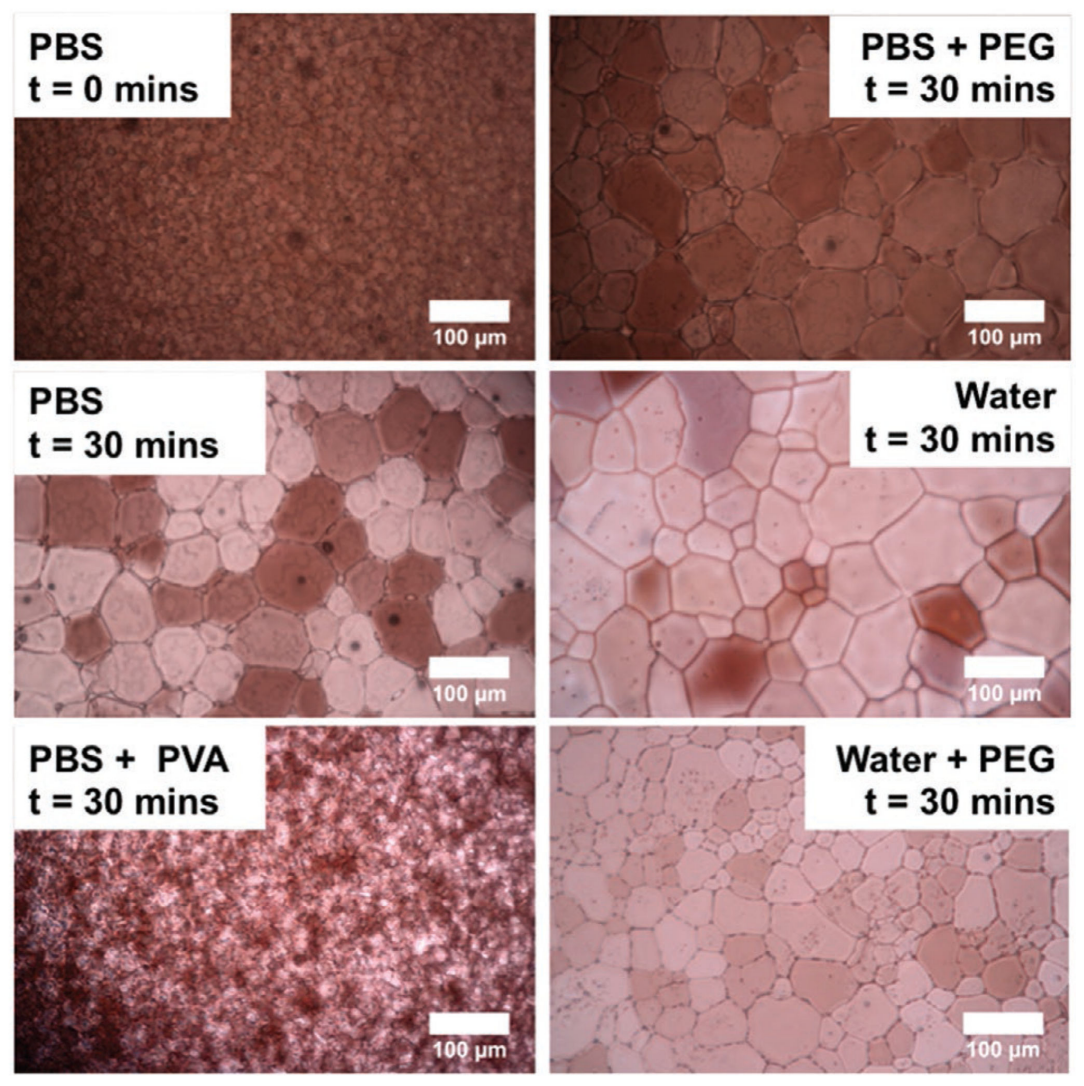
Example cryomicrographs of “splat” tests. Images are after the indicated period of time, following annealing at – 8 °C. PBS contains 0.137 m NaCl. PVA, poly(vinyl alcohol), *M*_n_ = 880 g mol^−1^; PEG, poly(ethylene glycol), *M*_n_ = 4000 g mol^−1^. Scale bar = 100 μm.

The first, and most widely used, method for measuring IRI is the “splat” assay as shown by Knight.^[26]^ This assay enables quick assessment of IRI activity by monitoring the growth of ice grains that have been nucleated rapidly at low temperatures (≈−80 °C) to ensure only growth (not nucleation) events are probed. This is a kinetic assay, hence shorter time points will always give smaller crystals. It is essential to compare materials under similar conditions to allow reliable assessment of more/less activity. In the present work, 30 min of annealing is used, as this is sufficient for screening, especially for identifying materials with lower or no activity, which quickly trend toward the control. More active IRIs (such as the antifreeze proteins) can inhibit ice growth for hours, but the majority of mimics are far less active than this, however all materials, no matter how active, will only slow ice growth, none can stop it completely. The temperature of the assay and the use of saline are also crucial. There must be sufficient NaCl (or another salt) to ensure a eutectic phase such that there is liquid water between the boundaries.^[26]^ False positives can occur when using pure water or too low saline, and hence even poly(ethylene glycol) (PEG) which is used as a negative control appears to inhibit recrystallization when conducted in water (see Supporting Information). The temperature is also crucial, with −6 to −8 °C often used, with lower temperatures potentially not allowing for recrystallization and yielding false positives. Data from these assays are often reported as the mean largest grain size (MLGS) or mean grain size (MGS). MLGS reports the largest grain in the field of view, and hence is slightly biased toward lower activity (more growth) and is easy to measure without image analysis software. MGS reports the average grain size in the field but requires measurement or counting of all crystals. Figure 1 shows example cryomicrographs of active (PVA) and inactive (PEG) polymers and shows the impact of conducting the assay in water, where a negative control can give the impression of activity. Ben et al. have reported domain recognition software to identify grain boundaries but it has not been widely used.^[27]^ An adaptation of this method uses concentrated sucrose solutions (>20 wt%) to generate isolated ice crystals. Due to the separation of crystals image analysis is facilitated. Using this method, Koop and co-workers have analyzed the kinetics of ice growth in the context of LSW theory to generate inhibition constants,^[25,28]^ but it is a slower method than the aforementioned “splat” assay, albeit one that provides detailed insight. Davies et al. have employed rapid freezing of capillaries to measure IRI, with the advantage that the samples can be stored for later use.^[29]^ However, this method is well suited for potent IRI active materials that inhibit all growth (as it is an end-point assay), so it is not suitable for those materials with weak activity as discussed here.

This article contains both new, and previously published IRI activity, focusing on that obtained by the “splat” assay where possible to allow critical comparison.

## 3 Proteins and Polymers Which Bind Ice

**Figure 2:**
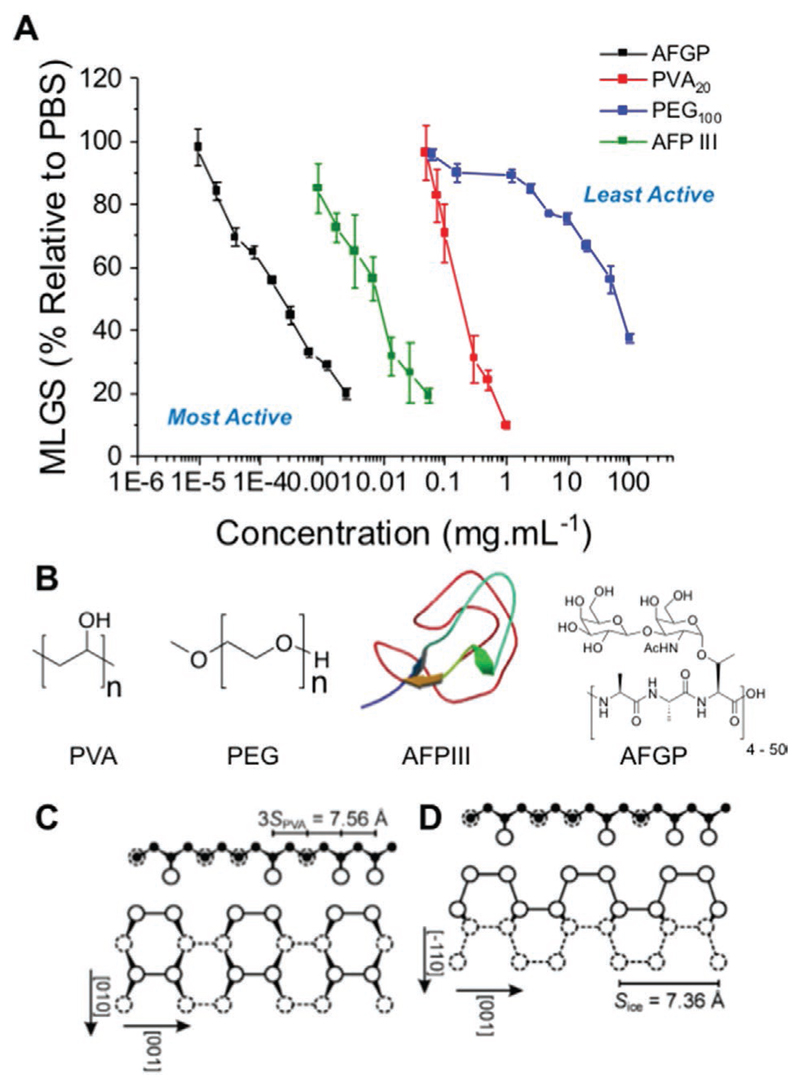
A) IRI activity of a range of highly active materials (PVA, AFPIII, AFGP) and PEG which is considered to be inactive. B) Structures of PVA, PEG, AFPIII, and AFGP. C) Proposed binding conformation for the adsorption of PVA onto the primary prism face (C), and secondary prism face (D). Reproduced with permission.^[36]^ Copyright 2006, Wiley.

To demonstrate the large range of IRI activity between some commonly used macromolecular inhibitors, the concentration dependence on the MLGS for AFGP_8_, AFP type III (AFPIII), PVA, and PEG (as a negative/weakly active) control is plotted in Figure 2. AFGP inhibits all growth at concentrations as low as 0.005 mg mL^−1^, but for PVA20 (one of the most active synthetic mimics reported^[4,24,30]^) up to 1 mg mL^−1^ is required—a 200-fold difference. The AFPIII (which is not glycosylated) showed activity between the two, inhibiting all growth at ≈0.1 mg mL^−1^. This difference in activity highlights the remarkable properties of AF(G)Ps and the immense challenge of reproducing their function. PEG, which is not reported to have any ice-binding/interacting effects, cannot fully inhibit growth even at 100 mg mL^−1^. This not only shows the vast range of potential IRI activities, but also highlights the key challenge in the discovery of useful new materials in that *any macromolecular material will slow ice growth if a sufficiently high concentration is added.*

This small set alone shows the importance of screening with a negative control, such as PEG, as essentially any material can claim to have IRI activity but the magnitude of this is the crucial parameter. A detailed discussion of AFGP/AFP’s high activity is beyond the scope of this feature article, but in both cases it appears to be due to specific recognition of the primary prism face of ice, which is the fastest growing face under standard conditions.^[31]^ There is still uncertainty as to how exactly AFGPs bind ice though, either through their glycans,^[32]^ or the peptide backbone.^[33]^ Hyperactive AFPs, which display a larger thermal hysteresis gap can also bind the basal face.^[34,35]^ Experimental and modeling evidence supports PVA’s unique activity being due to its hydroxyls precisely matching the spacing on the prism plane of ice, and a balance of enthalpy/entropy compensation leads to increased affinity and hence inhibition^[36–38]^ due to a multivalent or “zipper” effect (Figure 2C). This also explains why many other polyols fail to inhibit growth as they do not have this extended stretch of hydroxyls with precise spacing, with a range of glycopolymers or carbohydrates (see Supporting Information) showing very little activity.^[39,40]^ The molecular weight dependence of PVA’s activity has been explored in detail previously, and is outside the scope of this article.^[38,41]^ Polymer chain architecture has also shown to be important, with three-arm PVA demonstrating IRI activity equal to that of a linear polymer the same length as two of the arms.^[42]^ It has been suggested that this is because of the inability of the third arm to effectively bind a basal plane of ice, as the other two arms (binding in a linear fashion) confine the third.^[37]^

## 4 Ice-Binding Self-Assembled Materials

**Figure 3:**
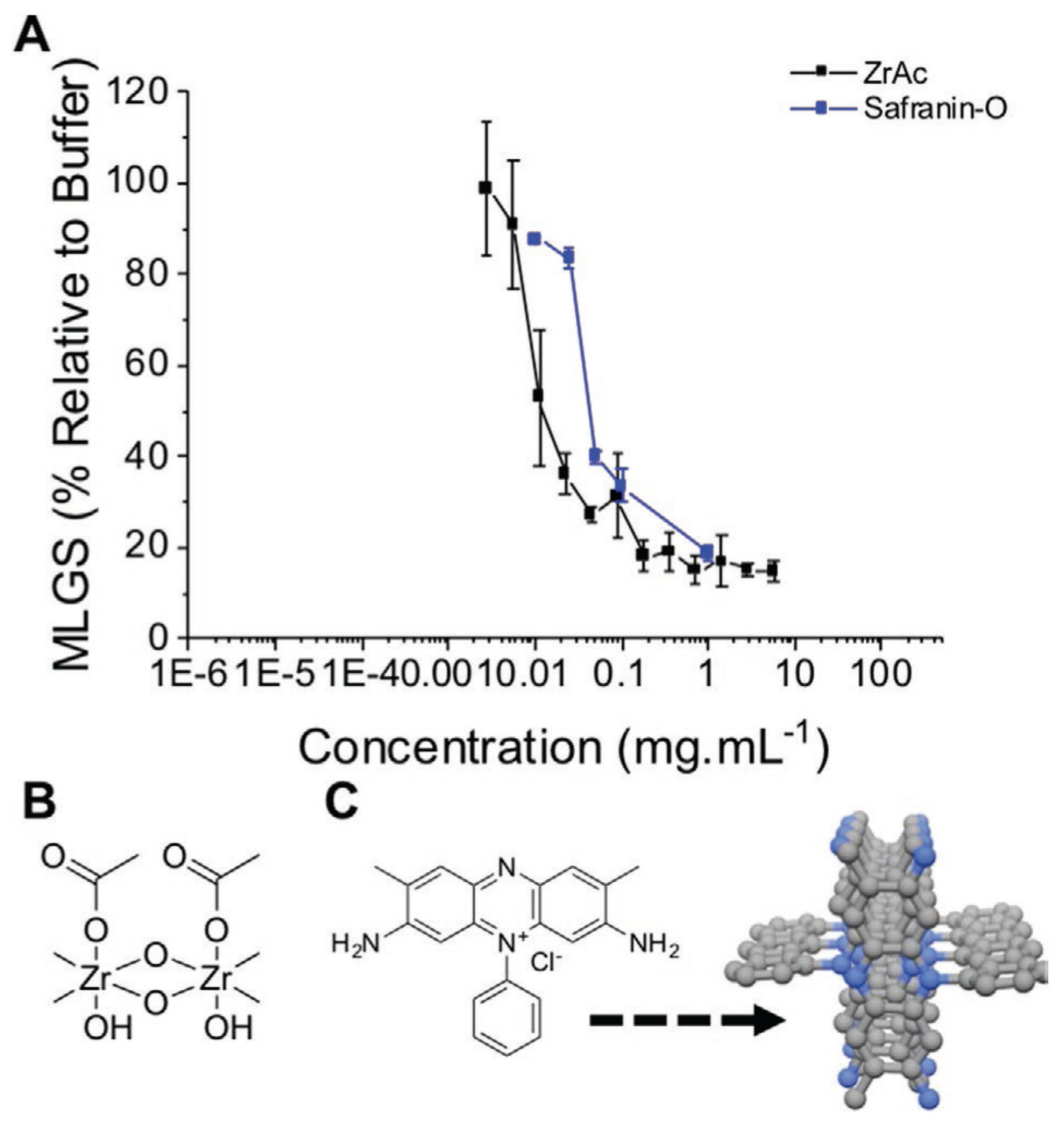
Supramolecular ice growth inhibitors. A) IRI comparison of ZrAc and safranin-O, B) Proposed ZrAc solution structure with acetate face, and hydroxyl face.^[45]^ C) Safranin-O self-assembly into fibers. Reprinted (adapted) with permission.^[43]^ Copyright 2016, American Chemical Society.

The previous section summarizes the activity range of materials with characterized (or hypothesized) ice-binding interactions. As an interesting example of unusual IRI active materials, Drori et al. showed that safranin-O (a dye) is a potent IRI (Figure 3).^[43]^ Safranin-O has no obvious structural similarities to AFPs but in aqueous solution self-assembles into fibers which can present a hydrophobic face that is similar to the solution structure of some AFPs. Safranin-O can inhibit all ice growth at just 1 mg mL^−1^, similar to PVA. Addition of a phenyl group disrupts the self-assembly and turns off the activity, supporting a mechanism where an extended hydrophobic face is essential.

In solution, zirconium acetate (ZrAc) can polymerize to form extended structures and it has been reported to have both ice shaping and IRI activity.^[44,45]^ In solution, the structure of ZrAc produces a coordination polymer with regularly spaced hydroxyls and acetate groups, which could either hydrogen bond to ice (e.g., like PVA) or interact via hydrophobic interactions (as with AFPs). Figure 3A shows ZrAc activity (note that this is relative to a pH 5 acetate buffer as it is not soluble in standard PBS) revealing it has similar activity to the AFP and safranin-O, inhibiting all growth below 1 mg mL^−1^. Other metal acetates, to the best of our knowledge, do not have this effect but it does support a hypothesis that the design of supramolecular IRIs is indeed possible, if not easy. There is also a report of graphene oxide inhibiting ice growth in this concentration range (all growth stopped at 5 mg mL^−1^).^[46]^ Graphene oxide is also known to affect ice nucleation^[47,48]^; such dual activity has also been observed for some AFPs.^[49]^

## 5 Facially Amphiphilic, Non-Ice-Binding Materials and Compounds

**Figure 4:**
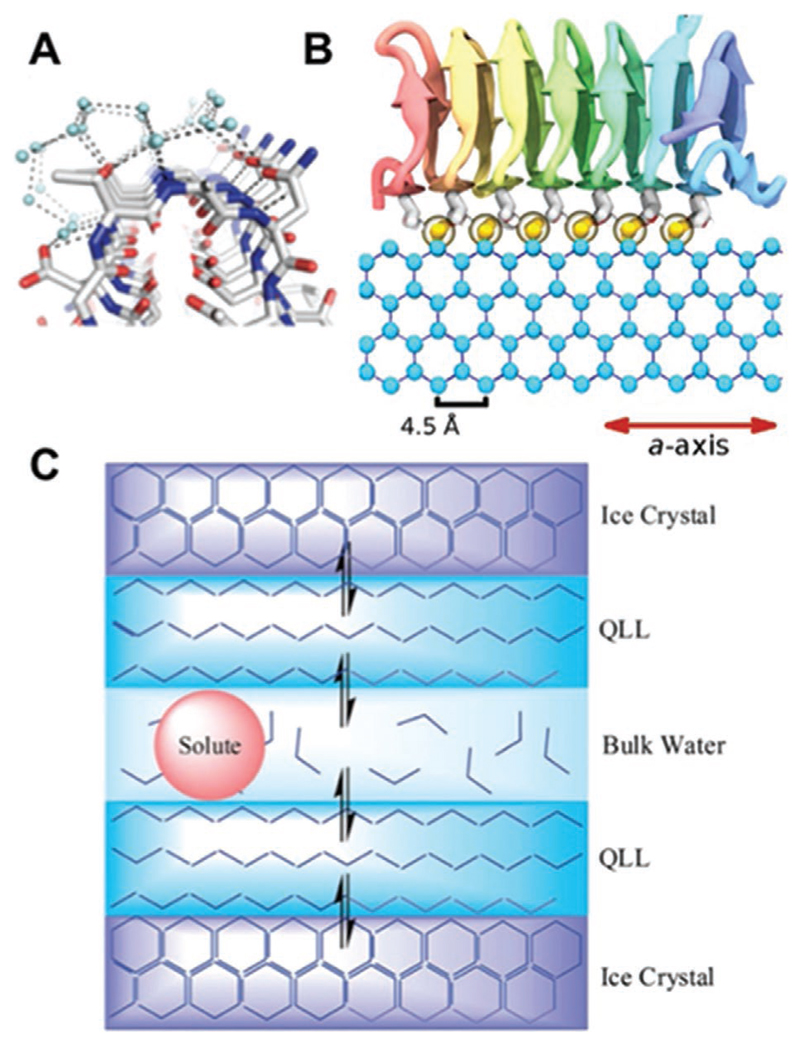
Modes of action resulting in IRI (and in case of A/B ice shaping). A) Anchored clathrate water on the surface of an antifreeze protein (*MpAFP_RIV*). Adapted with permission.^[53]^ Copyright 2011. National Academy of Sciences. B) Molecular dynamics results showing Spruce Budworm AFP with ordered water on AFP surface binding to the prism plane of ice. Reproduced with permission.^[54]^ Copyright 2015, eLife Sciences Publications. C) Interruption of the bulk water/semi-ordered water interface. Reproduced with permission.^[62]^ Copyright 2014, Royal Society of Chemistry. Note: QLL (quasi-liquid layer) is indicated in the reproduced figure, but the term semi-ordered layer is used here.

The previous examples are materials which have characterized ice-binding activity, either from direct measurements, or by observation of ice crystal shaping, and in most cases this affinity for the primary prism plane of ice is what drives their observable activity. In 2003, Ben et al. discovered that simplification of the AFGP structure to present only *α*-galactose (in place of the native disaccharide) and replacing alanine with glycine resulted in peptides that had definite IRI but no thermal hysteresis.^[19]^ This finding suggested that it was possible to achieve potent IRI, but without the need to target a specific plane of ice, that is, *there are multiple molecular level mechanisms which can give rise to the same macroscopic effect.* The same group has reported a series of modified glycopeptides with potent IRI, with the distance between backbone^[50]^ and glycan being important as well as backbone hydrophobicity.^[51,52]^ Figure 4A shows anchored clathrate water on the surface of an AFP, which directs the binding to multiple ice planes (basal and prism) due to crystallographic matches.^[53]^ Figure 4B shows a simulation of spruce budworm AFP and its hypothesized binding to the prism plane via coordinated water molecules providing a match.^[54]^ Whilst the molecular binding details are still under investigation, there is overwhelming evidence for AFPs binding to ice faces and strong evidence of molecular level interactions have been determined.^[34,55–59]^ However, for the facially amphipathic molecules, with no evidence for ice binding, an alternative molecular level mechanism which can give rise to IRI is required. One proposal is that these can sit at the semi-ordered water layer (sometimes referred to as quasi-liquid layer, QLL, which is strictly a definition at surfaces)^[60]^/bulk water interface, rather than directly interact with ice faces.^[61,62]^ If the compounds can disorder this region, they will not fit well with bulk water, nor semi-ordered water later and hence the barrier to adding more water to the ice crystal is higher and slows recrystallization.^[61]^

**Figure 5:**
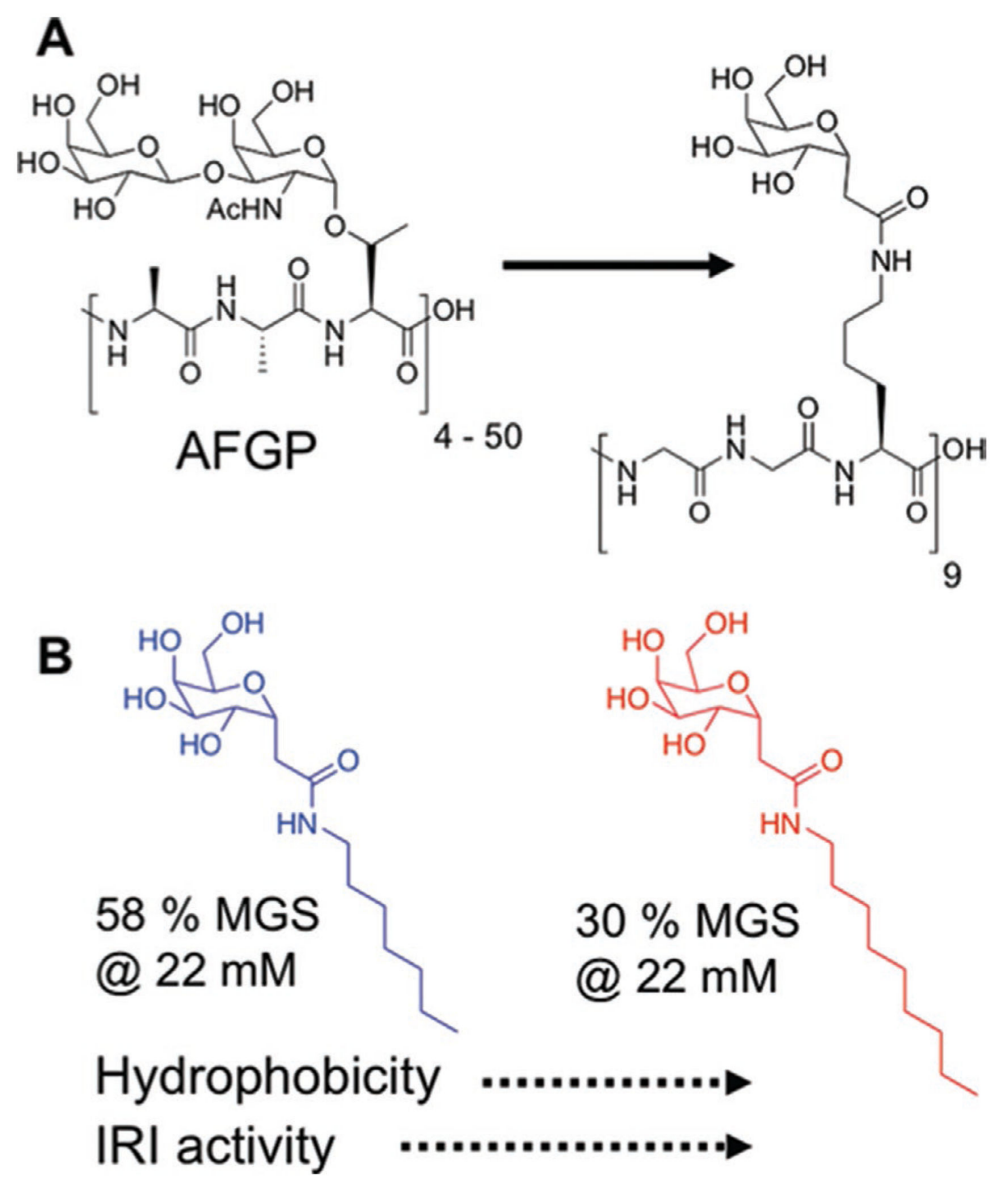
Glycomimetics of AFGPs. A) Structural simplifications to a glycopeptide with IRI but not thermal hysteresis activity. Reproduced with permission.^[19]^ Copyright 2003, Springer Nature Switzerland AG. B) Impact of increased hydrophobic tail length on observable IRI. Reproduced with permission.^[63]^ Copyright 2013, Royal Society of Chemistry.

Ben and co-workers synthesized a range of alkylated galactose derivatives and assessed their IRI. Increasing the alkyl chain length from seven to nine carbon atoms lead to an increase in activity (Figure 5) until micellization occurred. Micellization buries the hydrophobic units in the core and hence only hydrophilic units are presented, highlighting the delicate balance required in this approach to induce IRI.^[63]^ Lysine-based surfactants with increasing hydrophobic modifications show increased IRI activity, and clearly have no ice-binding site. These carry a net charge confirming that a sugar unit is not an essential moiety for IRI.^[64,65]^ It should be noted, however, that removing the glycan unit from an AFGP completely removes its ability to bind to ice effectively, as shown by Nishimura et al.,^[66]^ which demonstrates that conclusions drawn for one class of IRI may not be valid for others.

**Figure 6:**
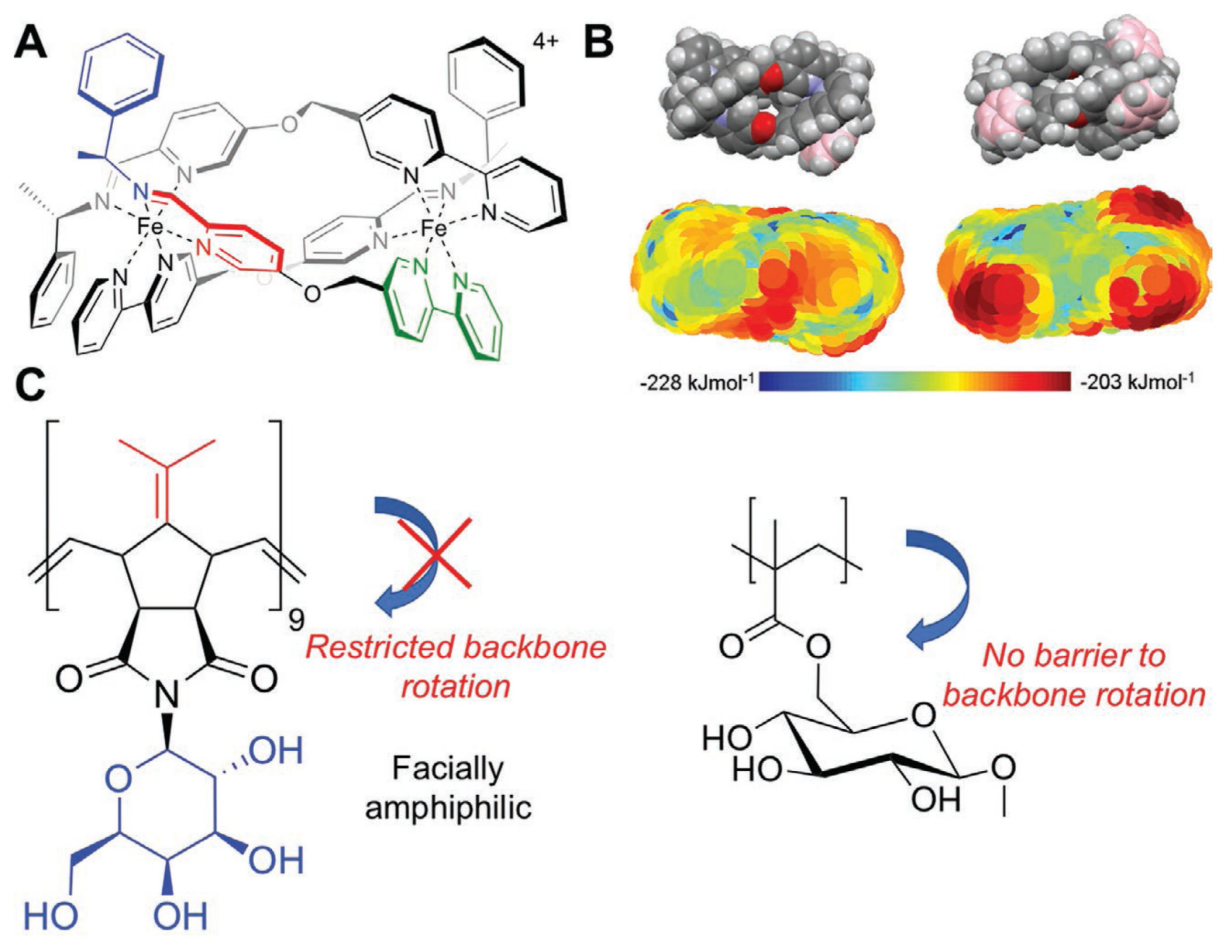
Facially amphiphilic IRI active compounds. A) Self-assembled metallohelices. B) Charge distribution and hydrophobicity plots, colored according to scale. Reproduced with permission.^[74]^ Copyright 2017, American Chemical Society. B) IRI active rigid glycopolymers (from ROMP) versus weakly active flexible glycopolymers (with saturated backbones).

To avoid the issue of aggregation upon sequential increases in hydrophobicity, Mitchell et al. exploited Nisin A as a pH switchable IRI. Nisin A is an antimicrobial peptide that upon lowering the pH assumes a facially amphipathic structure, with segregated hydrophobic/hydrophilic domains.^[67]^ Using the “splat” assay, Nisin shows IRI at low pH (or upon coordination of metal ions), which was erased at higher pHs. This provided more evidence for the hypothesis that the presentation of a spatially segregated hydrophobic face is crucial for activity. The actual IRI seen, however, was rather weak requiring 5 mg mL^−1^ to inhibit 50% of ice growth. AFPs and AFGPs also show facial amphiphilicity[66,68] and simulations suggest this facial amphiphilicity is crucial for AFGPs^[33]^ but not necessarily for AFPs.^[58,69]^ These experiments provided crucial data that a second structural feature, after ice binding, can be used to introduce IRI into a synthetic material; spatially segregated hydrophobic/hydrophilic domains, but without micellization. Mitchell et al. further used self-assembling metallohelices to probe this concept. The helices show patchy hydrophobicity and no obvious ice-binding sites, yet can inhibit ice growth completely at around 5 mg mL^−1^, making them one of the more potent synthetic inhibitors reported (Figure 6A,B). A key feature of AFGPs is that increasing their length (i.e., more tripeptide repeats) leads to more IRI (and TH) activity.^[25]^ Hence, synthetic polymers, where the chain length is easily tuned, are appealing based on this design principle.^[70]^ However, it is challenging to separate hydrophobic and hydrophilic domains when using conventional radical polymerization which gives flexible backbones. Tew et al. reported that using ROMP (ring opening metathesis polymerization) semi-rigid polymers can be obtained. ROMP-derived polymers have limited rotation due to the alkene (and often cycle) structure in the backbone, hence hydrophobic groups can be placed opposite hydrophilic, to install facial amphiphilicity.^[71,72]^ Graham et al. synthesized ROMP glycopolymers bearing a galactose residue on one face, and either an oxo or a fulvo (more hydrophobic) residue on the opposing face. IRI assays showed that the fulvo polymers were far more active than oxo, confirming that the amphiphilicity rather than the presence of a glycan is the key structural feature.^[73]^ It also supported the hypothesis that segregation of these groups is crucial. No ice shaping was seen for these materials, suggesting no ice binding.

## 6 Flexible Polymers and Hydrophobicity

**Figure 7:**
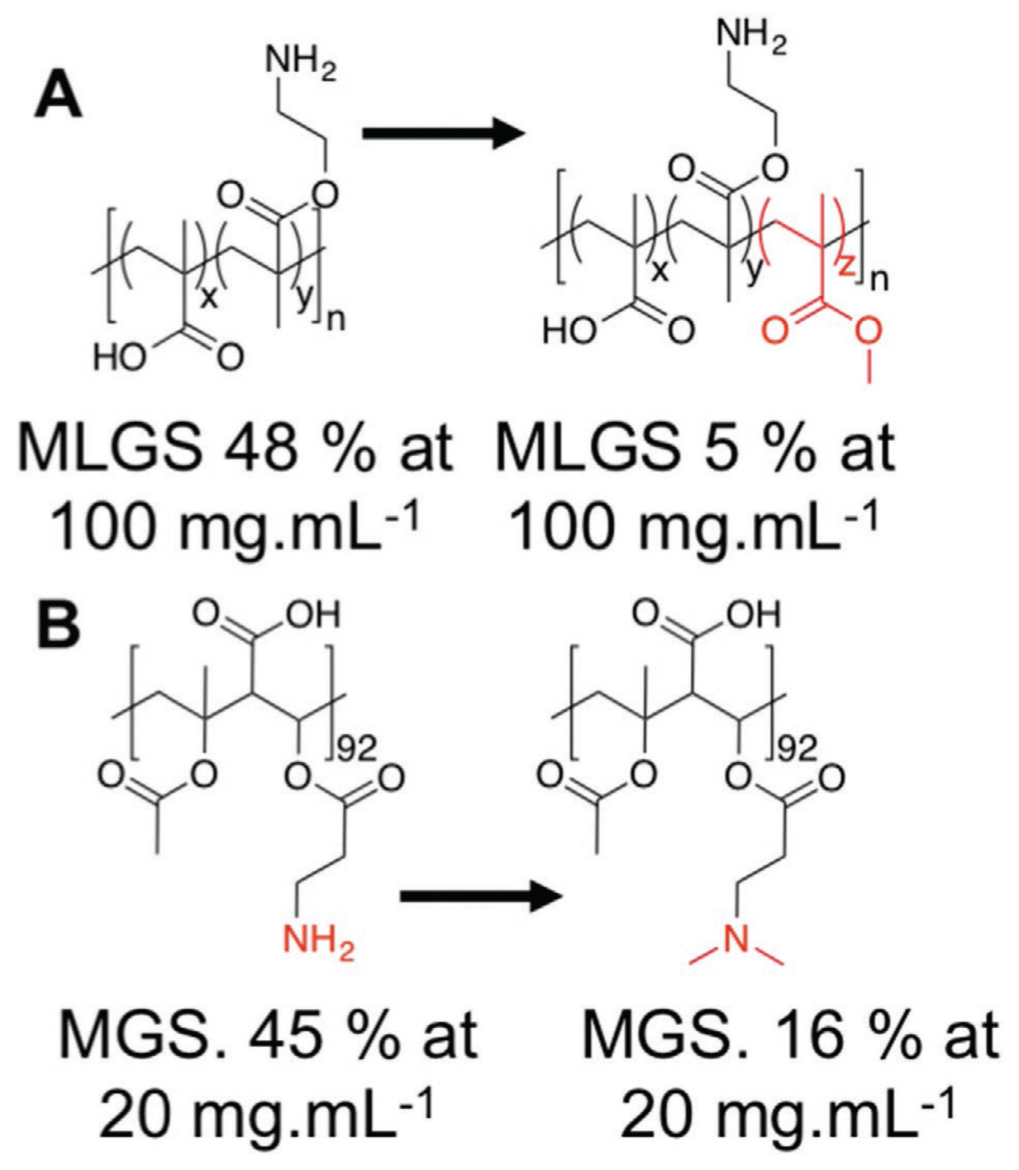
Poly(ampholyte)s with reported IRI activity. A) Random copolymers of methacrylic acid with aminoethyl methacrylate enhanced by addition of methyl methacrylate. Reproduced with permission.^[81]^ Copyright 2013, Informa UK Limited. B) Regioregular alternative copolymers derived from maleic anhydride and vinyl acetate. Reproduced with permission.^[79]^ Copyright 2017, American Chemical Society.

It is clear that correct 3D placement of hydrophobic groups can modulate IRI activity and may be crucial in natural AF(G)Ps, in addition to their remarkable ice-binding properties.^[2]^ However, in the case of synthetic polymers, most are flexible coils due to a saturated backbone with sp3 bonding, and cannot present specific “patches” of hydrophobicity. There has been interest in the introduction of IRI activity into polymers based on the statistical incorporation of hydrophobic groups, which may offer a route to low-cost IRI active materials. The most studied in this context are poly(ampholytes)—polymers bearing mixed positive and negative charges. Matsumura et al. reported that carboxylation of poly(*ε*-lysine), to give a 50:50 mix of charges, produced polymers that were potent cryopreservatives when used in conjugation with traditional cryoprotective agents.^[75,76]^ Later studies showed that this ratio of charges was essential for both cryopreservation and for IRI activity.^[77]^ Matsumura has investigated poly(ampholytes) derived from dimethylaminoethyl methacrylate (DMAEMA) and methacrylic acid (MAA), which have some IRI activity as well as being effective cellular cryoprotectants. Copolymers of DMAEMA and MAA achieved around 50% inhibition at 100 mg mL^−1^ (which is identical to PEG), however upon the incorporation of as little as 5% butyl methacrylate or octyl methacrylate into the chain, a MGS of 5% was observed at the same concentration, suggesting an increase in activity Figure 7.^[78]^ To study this in more detail, Stubbs et al. synthesized a panel of regioregular poly(ampholyte)s using maleic anhydride copolymers, which ensure an alternating, rather than random, copolymer structure. Sequential increases in hydrophobicity were introduced by ring opening of the anhydride using alcohols.^[79]^ There was a clear trend that increased hydrophobicity increased the IRI activity. It is crucial to note here that the magnitude of activity is far less than the previous classes (AF(G)Ps/PVA) with some ice growth still occurring at 20 mg mL^−1^, hence care must be taken when discussing potency. These results, when taken together, suggest that while hydrophobicity is important, the spatial segregation of these domains needs to be carefully controlled to access IRI active materials. Similar observations have been made by copolymerization of vinyl pyrrolidone with increasingly hydrophobic units. Homo poly(vinyl pyrrolidone) has essentially no activity but incorporation of hydrophobic units did increase IRI, albeit at a level which was only just above that of PEG.^[80]^

### 6.1 Summary

**Figure 8:**
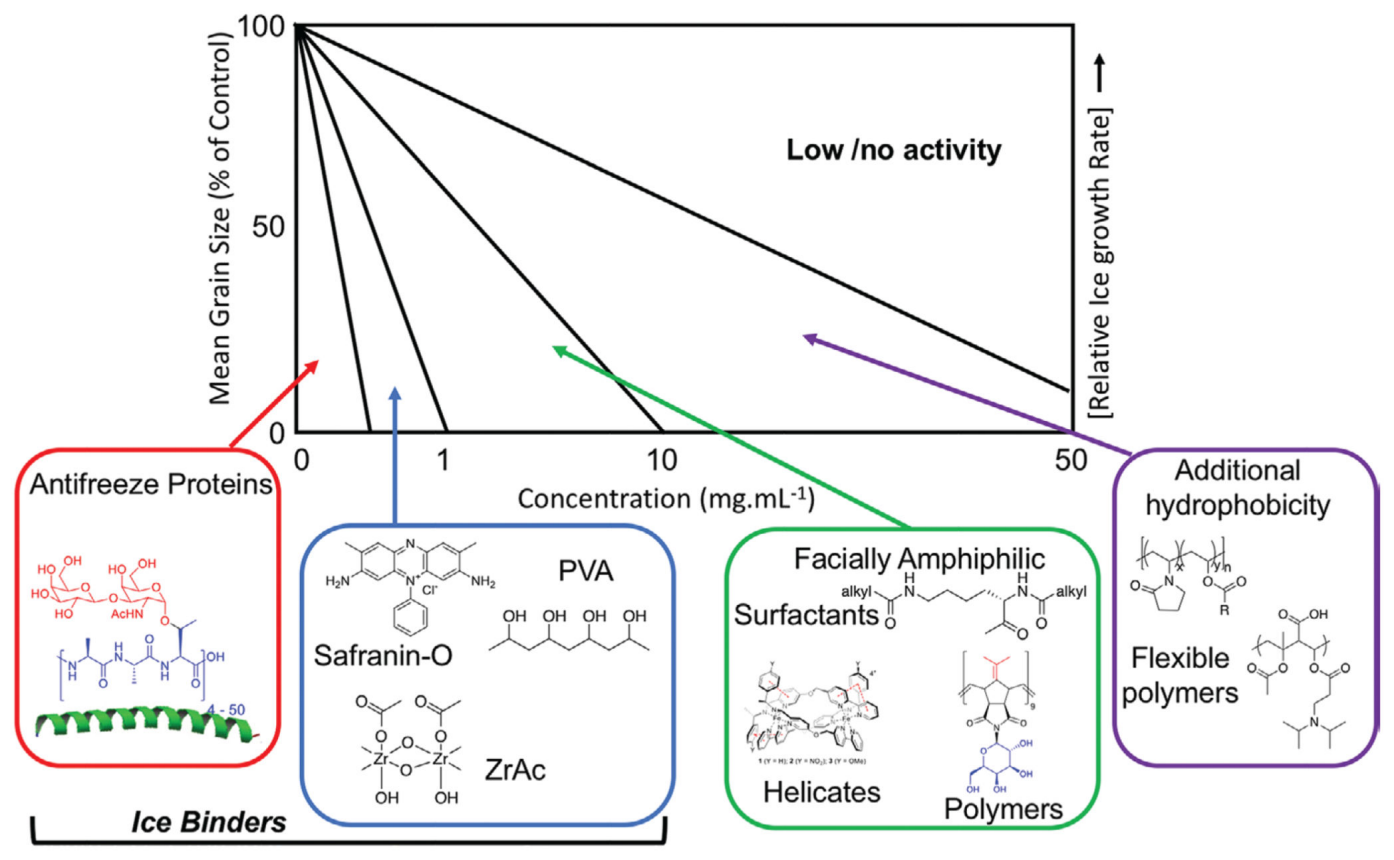
Summary of range of activities observed for different classes of IRI active compounds. It is important to note that the size indication is based on static 30 min measurement, and that in all cases the ice crystals keep growing and hence is a snapshot of a rate of growth.

Considering the examples shown above, we propose a rough guide for activity considerations when evaluating a new material for IRI, especially those which are far less active than AF(G) Ps Figure 8. We divide the materials into approximately four zones, which may align (based on our current understanding) with their underlying mechanism of action, as well as magnitude of activity. The most active are AF(G)Ps, which function at sub 0.1 mg mL^−1^ levels. There is also significant evidence for their selective and strong binding to specific planes of ice, which is crucial for their activity. Next, we include those that inhibit in the range up to ≈1 mg mL^−1^, which includes PVA, ZrAc, and safranin-O. These materials also shape ice and have potent IRI activity, with at least some of their activity arising from ice-face binding (if not all). After this, there are the materials that have no evidence for ice binding (at the moment) but are still relatively potent. It is crucial to note that ice binding could be occurring, but that it is so weak or transient that macroscopic ice shaping is not seen. However, these materials are facially amphiphilic with segregation of the domains crucial for their function, enabling them to slow ice growth fully, typically in a range <10 mg mL^−1^. It should be highlighted that these are approximate definitions, for example, the highly active lysine surfactants of Ben^[63]^ have similar activity to the ice binders; hence the need for appreciation that IRI is a continuum not a binary property. Next is the broad zone of “weak inhibitors,” which includes poly(ampholyte)s and hydrophobically modified polymers, with the upper boundary being the activity of PEG. Materials in this range do not appear to bind ice to any appreciable extent and in many cases cannot completely inhibit ice growth over any time period. Nonetheless, that does not rule out potential applications, where slowing ice is crucial or where high concentrations can be applied.

We feel this (simplistic) display provides a framework to discuss new inhibitors, especially those where ultra-high concentrations of up to 100 mg mL^−1^ have been tested,^[81]^ which is clearly not a specific effect. There is no suggestion here as to which region is worthy of the most study, nor which is most promising for a specific application, but rather to help as a guide in defining materials’ relative function, and to ensure that false positives due to simple colligative effects are not misreported in the literature as the field grows.

## 7 Conclusions

In conclusion, we present a concise evaluation of a range of IRI active macromolecules covering several orders of magnitude in terms of activity. We show that even though AFGPs and AFPs are clearly far more active than synthetic mimics, their role in cryopreservation is not just linked to absolute activity but also availability, cost, and possible toxicity/immunogenicity. We propose a grouping of materials into different activity magnitudes especially at the lower end of the activity scale (see Section 6.1), and highlight that this is not an on/off property but rather a continuum. Thus, activity needs to be defined relative to a negative control, to place it in context. We also propose that the less active materials seem to be able to function due to their amphipathic structure, and can be more active due to a precise interaction with ice.

## 8 Experimental Section

### Ice Recrystallization Inhibition “Splat” Assay

A 10 μL droplet of the macromolecule in PBS solution was dropped from 1.4 m onto a glass microscope coverslip, which is on top of an aluminum plate cooled to −78 °C using dry ice. The droplet freezes instantly upon impact with the plate, spreading out and forming a thin wafer of ice. This wafer is then placed on a liquid nitrogen cooled cryostage held at −8 °C. The wafer is then left to anneal for 30 min at −8 °C. Three micrographs were taken of the wafer and the longest grain crystals as well as the total number of crystals in the image are counted using ImageJ, and reported as a percentage of area compared to PBS control.

## Supplementary Material

Supporting Information is available from the Wiley Online Library or from the author.

Supporting information

## References

[1] Davies PL, Sykes BD (1997). Curr Opin Struct Biol.

[2] Davies PL (2014). Trends Biochem Sci.

[3] Harding MM, Anderberg PI, Haymet ADJ (2003). Eur J Biochem.

[4] Voets IK (2017). Soft Matter.

[5] Valarezo WO, Lynch FT, McGhee RJ (1993). J Aircr.

[6] Parent O, Ilinca A (2011). Cold Reg Sci Technol.

[7] Brockbank K, Taylor M (2007). Adv Biopreserv.

[8] John Morris G, Acton E (2013). Cryobiology.

[9] Mazur P (1970). Science.

[10] Mazur P, Fuller B, Lane N, Benson EE (2004). Life Frozen State.

[11] Song YC, Khirabadi BS, Lightfoot F, Brockbank KGM, Taylor MJ (2000). Nat Biotechnol.

[12] Polge C, Smith AU, Parkes AS (1949). Nature.

[13] Wowk B, Leitl E, Rasch CM, Mesbah-Karimi N, Harris SB, Fahy GM (2000). Cryobiology.

[14] Carpenter JF, Hansen TN (1992). Proc Natl Acad Sci USA.

[15] Chao H, Davies PL, Carpenter JF (1996). J Exp Biol.

[16] Matsumoto S, Matsusita M, Morita T, Kamachi H, Tsukiyama S, Furukawa Y, Koshida S, Tachibana Y, Nishimura SI, Todo S (2006). Cryobiology.

[17] Tomczak MM, Hincha DK, Estrada SD, Feeney RE, Crowe JH (2001). Biochim Biophys Acta, Biomembr.

[18] Rubinsky B, Arav A, Mattioli M, Devries AL (1990). Biochem Biophys Res Commun.

[19] Eniade A, Purushotham M, Ben RN, Wang JB, Horwath K (2003). Cell Biochem Biophys.

[20] Mitchell DE, Fayter AER, Deller RC, Hasan M, Gutierrez-Marcos J, Gibson MI (2019). Mater Horiz.

[21] Deller RC, Vatish M, Mitchell DA, Gibson MI (2014). Nat Commun.

[22] Capicciotti CJ, Poisson JS, Boddy CN, Ben RN (2015). Cryobiology.

[23] Briard JG, Poisson JS, Turner TR, Capicciotti CJ, Acker JP, Ben RN (2016). Sci Rep.

[24] Biggs CI, Bailey TL, Ben Graham, Stubbs C, Fayter A, Gibson MI (2017). Nat Commun.

[25] Budke C, Dreyer A, Jaeger J, Gimpel K, Berkemeier T, Bonin AS, Nagel L, Plattner C, Devries AL, Sewald N, Koop T (2014). Cryst Growth Des.

[26] Knight CA, Wen D, Laursen RA (1995). Cryobiology.

[27] Pezacki JP, Noestheden M, Ben RN, Jackman J, Moffat D, Findlay S (2007). Biochem Biophys Res Commun.

[28] Olijve LLC, Oude Vrielink AS, Voets IK (2016). Cryst Growth Des.

[29] Tomczak MM, Marshall CB, Gilbert JA, Davies PL (2003). Biochem Biophys Res Commun.

[30] He Z, Liu K, Wang J (2018). Acc Chem Res.

[31] Rahman AT, Arai T, Yamauchi A, Miura A, Kondo H, Ohyama Y, Tsuda S (2019). Sci Rep.

[32] Meister K, DeVries AL, Bakker HJ, Drori R (2018). J Am Chem Soc.

[33] Mochizuki K, Molinero V (2018). J Am Chem Soc.

[34] Scotter AJ, Marshall CB, Graham LA, Gilbert JA, Garnham CP, Davies PL (2006). Cryobiology.

[35] Marshall CB, Fletcher GL, Davies PL (2004). Nature.

[36] Budke C, Koop T (2006). ChemPhysChem.

[37] Naullage PM, Lupi L, Molinero V (2017). J Phys Chem C.

[38] Congdon T, Notman R, Gibson MI (2013). Biomacromolecules.

[39] Gibson MI, Barker CA, Spain SG, Albertin L, Cameron NR (2009). Biomacromolecules.

[40] Deller RC, Congdon T, Sahid MA, Morgan M, Vatish M, Mitchell DA, Notman R, Gibson MI (2013). Biomater Sci.

[41] Vail NS, Stubbs C, Biggs CI, Gibson MI (2017). ACS Macro Lett.

[42] Congdon TR, Notman R, Gibson MI (2017). Eur Polym J.

[43] Drori R, Li C, Hu C, Raiteri P, Rohl AL, Ward MD, Kahr B (2016). J Am Chem Soc.

[44] Deville S, Viazzi C, Leloup J, Lasalle A, Guizard C, Maire E, Adrien J, Gremillard L (2011). PLoS One.

[45] Mizrahy O, Bar-Dolev M, Guy S, Braslavsky I (2013). PLoS One.

[46] Geng H, Liu X, Shi G, Bai G, Ma J, Chen J, Wu Z, Song Y, Fang H, Wang J (2017). Angew Chem Int Ed.

[47] Whale TF, Rosillo-Lopez M, Murray BJ, Salzmann CG (2015). J Phys Chem Lett.

[48] Biggs CI, Packer C, Hindmarsh S, Walker M, Wilson NR, Rourke JP, Gibson MI (2017). Phys Chem Chem Phys.

[49] Wilson PW, Osterday KE, Heneghan AF, Haymet ADJ (2010). J Biol Chem.

[50] Liu S, Ben RN (2005). Org Lett.

[51] Balcerzak AK, Capicciotti CJ, Briard JG, Ben RN (2014). RSC Adv.

[52] Capicciotti CJ, Trant JF, Leclère M, Ben RN (2011). Bioconjugate Chem.

[53] Garnham CP, Campbell RL, Davies PL (2011). Proc Natl Acad Sci USA.

[54] Kuiper MJ, Morton CJ, Abraham SE, Gray-Weale A (2015). eLife.

[55] Daley ME, Spyracopoulos L, Jia Z, Davies PL, Sykes BD (2002). Biochemistry.

[56] Olijve LLC, Meister K, Devries AL, Duman JG, Guo S, Bakker HJ (2016). Proc Natl Acad Sci USA.

[57] Hudait A, Moberg DR, Qiu Y, Odendahl N, Paesani F, Molinero V (2018). Proc Natl Acad Sci USA.

[58] Hudait A, Odendahl N, Qiu Y, Paesani F, Molinero V (2018). J Am Chem Soc.

[59] Qiu Y, Hudait A, Molinero V (2019). J Am Chem Soc.

[60] Asakawa H, Sazaki G, Nagashima K, Nakatsubo S, Furukawa Y (2016). Proc Natl Acad Sci USA.

[61] Tam RY, Ferreira SS, Czechura P, Ben RN, Chaytor JL (2008). J Am Chem Soc.

[62] Balcerzak AK, Capicciotti CJ, Briard JG, Ben RN (2014). RSC Adv.

[63] Trant JF, Biggs RA, Capicciotti CJ, Ben RN (2013). RSC Adv.

[64] Balcerzak AK, Febbraro M, Ben RN (2013). RSC Adv.

[65] Capicciotti CJ, Leclère M, Perras FA, Bryce DL, Paulin H, Harden J, Liu Y, Ben RN (2012). Chem Sci.

[66] Tachibana Y, Fletcher GL, Fujitani N, Tsuda S, Monde K, Nishimura SI (2004). Angew Chem Int Ed.

[67] Mitchell DE, Gibson MI (2015). Biomacromolecules.

[68] Hakim A, Nguyen JB, Basu K, Zhu DF, Thakral D, Davies PL, Isaacs FJ, Modis Y, Meng W (2013). J Biol Chem.

[69] Naullage PM, Qiu Y, Molinero V (2018). J Phys Chem Lett.

[70] Gibson MIMI (2010). Polym Chem.

[71] Tew GN, Scott RW, Klein ML, Degrado WF (2010). Acc Chem Res.

[72] Ilker MF, Nüsslein K, Tew GN, Coughlin EB (2004). J Am Chem Soc.

[73] Graham B, Fayter AER, Houston JE, Evans RC, Gibson MI (2018). J Am Chem Soc.

[74] Mitchell DE, Clarkson G, Fox DJ, Vipond RA, Scott P, Gibson MI (2017). J Am Chem Soc.

[75] Matsumura K, Hyon S-HH (2009). Biomaterials.

[76] Mitchell DE, Cameron NR, Gibson MI (2015). Chem Commun.

[77] Mitchell DE, Lilliman M, Spain SG, Gibson MI (2014). Biomater Sci.

[78] Rajan R, Hayashi F, Nagashima T, Matsumura K (2016). Biomacromolecules.

[79] Stubbs C, Lipecki J, Gibson MI (2017). Biomacromolecules.

[80] Stubbs C, Congdon TR, Gibson MI (2019). Eur Polym J.

[81] Rajan R, Jain M, Matsumura K (2013). J Biomater Sci Polym Ed.

[82] Congdon T, Shaw P, Gibson MIMI (2015). Polym Chem.

