# Supplementary material for: Mimicking the Ice Recrystallization Activity of Biological Antifreezes. When is a New Polymer “Active”?
Source: Macromol Biosci. Author manuscript; Available in PMC 2019 Nov 4. (PMC6828557; doi:10.1002/mabi.201900082)
Supplement: Supporting information [file EMS84735-supplement-Supporting_information.pdf]

Copyright WILEY-VCH Verlag GmbH & Co. KGaA, 69469 Weinheim, Germany, 2019.

Supporting Information

for *Macromol. Biosci.*, DOI: 10.1002/mabi.201900082

Mimicking the Ice Recrystallization Activity of Biological Antifreezes. When is a New Polymer "Active"?

Caroline I. Biggs, Christopher Stubbs, Ben Graham, Alice E. R. Fayter, Muhammad Hasan, and Matthew I. Gibson*

Copyright WILEY-VCH Verlag GmbH & Co. KGaA, 69469 Weinheim, Germany, 2019.

Supporting Information

Mimicking the Ice Recrystallization Activity of Biological Antifreezes. When is a New Polymer 'Active'?

Caroline I. Biggs, Christopher Stubbs, Ben Graham, Alice E. R. Fayter, Muhammad Hasan Matthew I. Gibson*

Experimental Section

Materials

Poly(ethylene glycol) (PEG) (4kDa) and Safranin-O, phenosafranin, NaCl and ampicillin were purchased from Sigma-Aldrich and used as supplied unless otherwise stated. Poly(vinyl alcohol) (PVA_{20}) (800 Da) was synthesised as previously reported.[82] AFGP8 was provided by A. L. DeVries, University of Illinois at Urbana-Champaign, USA.

Physical and analytical Methods

Samples for western blot analysis were resolved on a polyacrylamide gel, transferred to a membrane and detected a using primary (monoclonal anti-polyhistidine) antibody and a secondary (goat anti-mouse IgG (H+L)) antibody. Fast protein liquid chromatography (FPLC) was performed using AKTA pure (GE Healthcare) with a flow rate of 1 mL.min^{-1} using PBS buffer. A Linkam Biological Cryostage BCS196 with T95-Linkpad system controller equipped with a LNP95-Liquid nitrogen cooling pump, using liquid nitrogen as the coolant (Linkam Scientific Instruments UK, Surrey, U.K.) was used to anneal ice wafers. An Olympus CX41 microscope equipped with a UIS-2 20x/0.45/∞/0−2/FN22 lens (Olympus Ltd., Southend on sea,

U.K.) and a Canon EOS 500D SLR digital camera was used to obtain all images. Image processing was performed using ImageJ, which is freely available from http://imagej.nih.gov/ij/.

Protein Expression

AFPIII expression and purification

A plasmid encoding for a hexahistidine-tagged AFPIII (T7, pET21b, P19614) was transformed into competent *Escherichia coli* TOP10 cells (New England Biolabs). Single colonies were selected and grown overnight in 10 mL Lysogeny broth (LB)-medium containing 100 μg.mL-1 ampicillin under continuous shaking (37 °C, 180 rpm). Preculture was added (40 mL in 1 L) to LB-medium with ampicillin and grown until OD600 = 0.6. Isopropyl β-D-1-thiogalactopyranoside (IPTG) was then added to the cells to a final concentration of 0.4 mM to induce protein expression overnight (16 °C, 180 rpm). The cells were harvested by centrifugation (4 °C, 5000 g, 10 minutes), the supernatant decanted and the cells resuspended in prechilled phosphate buffered saline (PBS) (7 mL, (>18.2 Ω mean resistivity, [NaCl] = 0.138 M, [KCl] = 0.0027 M, and pH 7.4)). Pierce protease inhibitor mini-tablets were added to the suspension and it was passed through a STANSTED 'Pressure Cell' FGP12800 homogeniser to undergo lysis. Bugbuster was added (500 μL) and the sample left spinning for 20 minutes. The cell lysate was centrifuged (4 °C, 40,000 g, 45 minutes) and the supernatant syringe filtered (0.2 μm) and passed through a pre-equilibrated (20 mL PBS) IMAC Sepharose 6 Fast Flow (GE Healthcare) column charged with Ni(II) ions. The column was washed first with PBS, then with 3 column volumes of 30 mM imidazole in PBS. 300 mM imidazole in PBS was used to elute bound AFP III and the protein purified via fast protein liquid chromatography (FPLC). Western blot and SDS-PAGE gel electrophoresis were used to identify AFP III, and the protein concentration determined using Thermo Scientific Pierce BCA assay kit and verified by measuring absorbance at 280 nm and using beer lambert law.

AFPI expression and purification

A plasmid encoding for a Thioredoxin (Trx) and hexahistidine-tagged bpAFPI was transformed into competent *Escherichia coli* RIPL cells (New England Biolabs). A colony was selected to inoculate 10 mL of LB-medium containing ampicillin (10 μL) and chlorophenicol (10 μL) and was grown overnight under continuous shaking (37 °C, 180 rpm). The following day, 20 mL of the preculture was added to 500 mL of LB-medium in a 2 L Erlenmeyer flask containing 500 μL of both antibiotics and grown at 37 °C until an OD_{600} of 0.6 was reached. The temperature was reduced to 16 °C and IPTG was added to a final concentration of 0.2 mM. The overexpression of the protein took place overnight under continuous shaking (180 rpm), following which the cells were centrifuged at 4000 rpm for 15 minutes at 4 °C. The pelleted cells were resuspended in 7 mL PBS and centrifuged in falcon tubes at 10,000 rpm for 10 minutes.

Each pellet was resuspended in lysis buffer (20 mM Tris-HCL, 150 mM NaCl) and passed through a STANSTED 'Pressure Cell' FPG12800 homogeniser in order to lyse the cells. Bugbuster (100 uL) was added to the cell lysate, following which it was centrifuged (4 °C, 20,000 rpm, 30 minutes) and the supernatant syringe filtered (0.2 μm) and passed through a pre-equilibrated (20 mL PBS) IMAC Sepharose 6 Fast Flow (GE Healthcare) column charged with Ni(II) ions. 5 mM imidazole was added to the column and left to equilibrate for 1 hour. The column was washed first with 100 mL of 30 mM imidazole in PBS, then the Ttx-tagged bpAFPI was eluted with 300 mM imidazole in PBS.

Tobacco Etch Virus (TEV) protease was added to the eluted Ttx-tagged bpAFPI protein to cleave the Trx tag. This solution was then purified by HPLC. Identification of AFPI was performed via QTOF mass spectrometry and SDS-PAGE gel electrophoresis; the cells were centrifuged (4 °C, 6000 RPM, 5 minutes) and the supernatant removed. 2x SDS loading dye

(25 µL) was added to aliquots from different stages of purification as well as whole cells (25 µL) and incubated (80 °C, 10 minutes). 15 µL of individual samples were loaded onto the gel and the gel ran at 200 V for 30 minutes. Coomassie blue (20 mL) was added to stain the gels for analysis.

Additional Data

The relative activity of AFPI, AFPII and AFGP assessed by both MGS and MLGS is shown below for comparison.

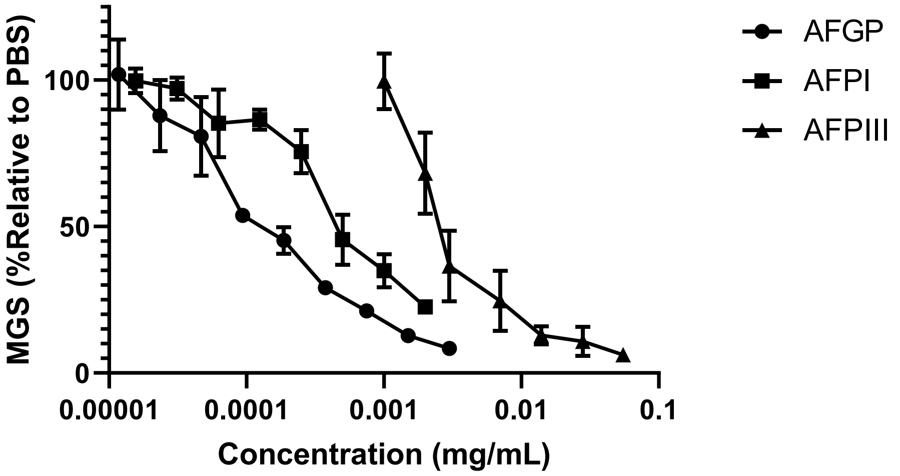

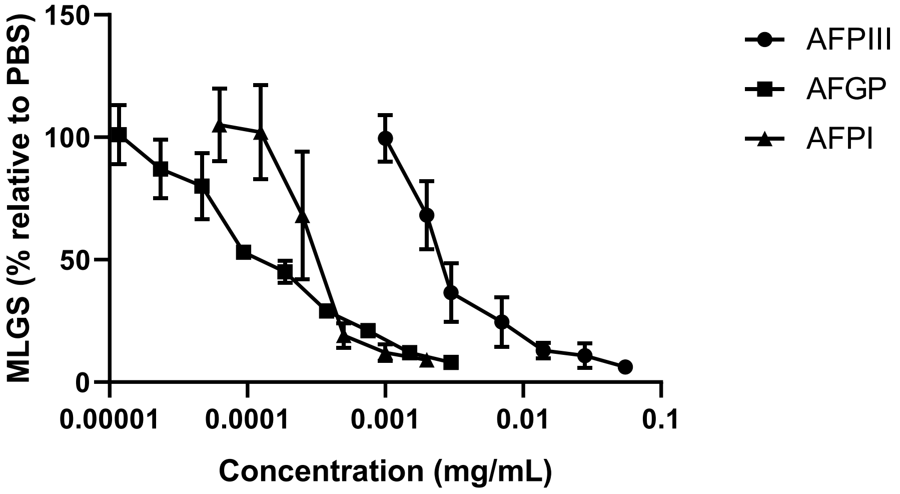

Figure S1. Mean largest grain size (MLGS) and mean grain size (MGS) comparison for a series of antifreeze proteins.

To highlight how average grain size and mean largest grain size correlate, data from a previously published paper from our group using poly(ampholytes) is shown in both formats .[79] MLGS is biased towards less activity for lesser active materials, but MGS is not, enabling a broader range of activities to be probed.

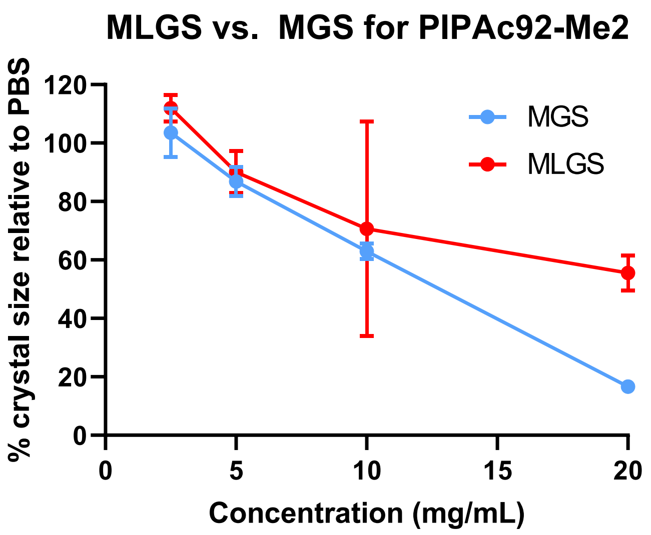

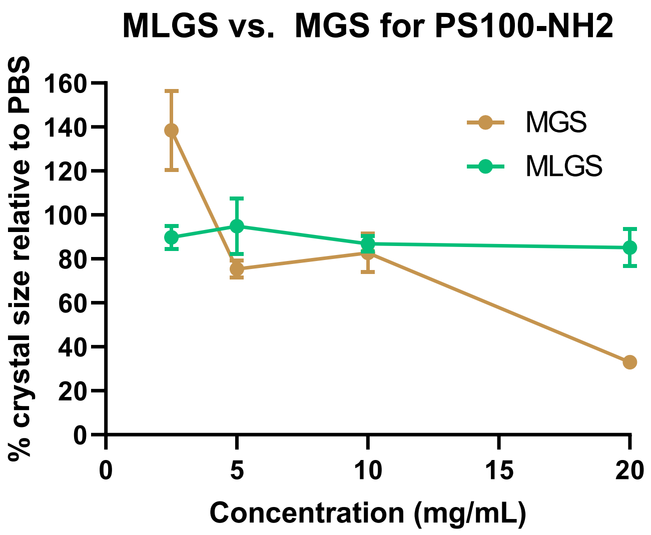

Figure S2. Comparison of MLGS verses MGS for two different poly(ampholytes). PIPAc92-Me2 is a Poly(isopropenyl acetate-*alt*-maleic anhydride) copolymer functionalised with dimethylamino ethanol (M_n unfunctionalised copolymer = 18,200 Da, Đ from SEC = 1.70). PS100-NH_2 is a Poly(styrene-*alt*-maleic anhydride) copolymer functionalised with ethanolamine (M_n unfuctionalised copolymer = 20,300 Da, Đ from SEC = 1.11) .

False positives due to solvent conditions.

As discussed in the main text, the ‘splat’ assay requires saline to be present to generate a eutectic phase and avoid false positives. Below is data (both as MGS and MLGS) for PEG, which is considered to be IRI inactive. As can be seen, espeically in MGS, splat tests conducted on PEG in water alone do limit ice growth, as a false positive and hence care must be taken, Figure S4. Figure S5 shows example micrographs to highlight this point.

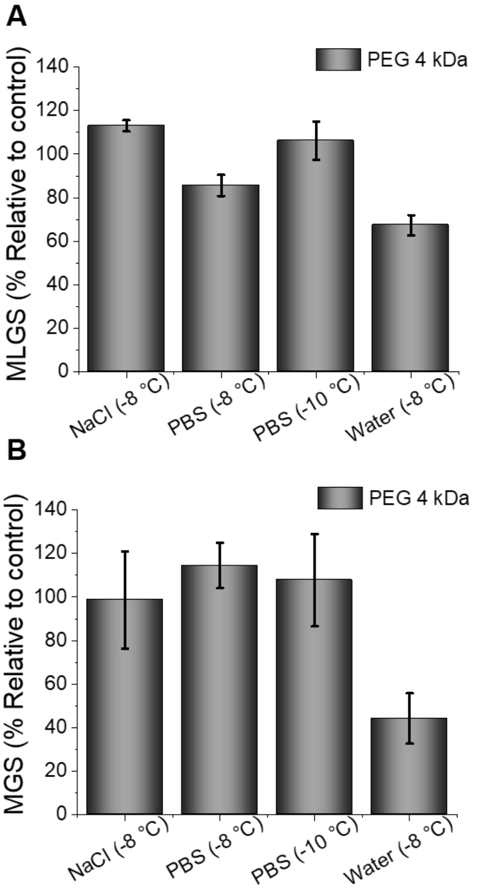

Figure S3. Comparison of MLGS verses MGS for PEG (M_n = 4,000 $g.mol^{-1}$) in saline, PBS, and pure water, at varying temperatures following 30 minutes annealing.

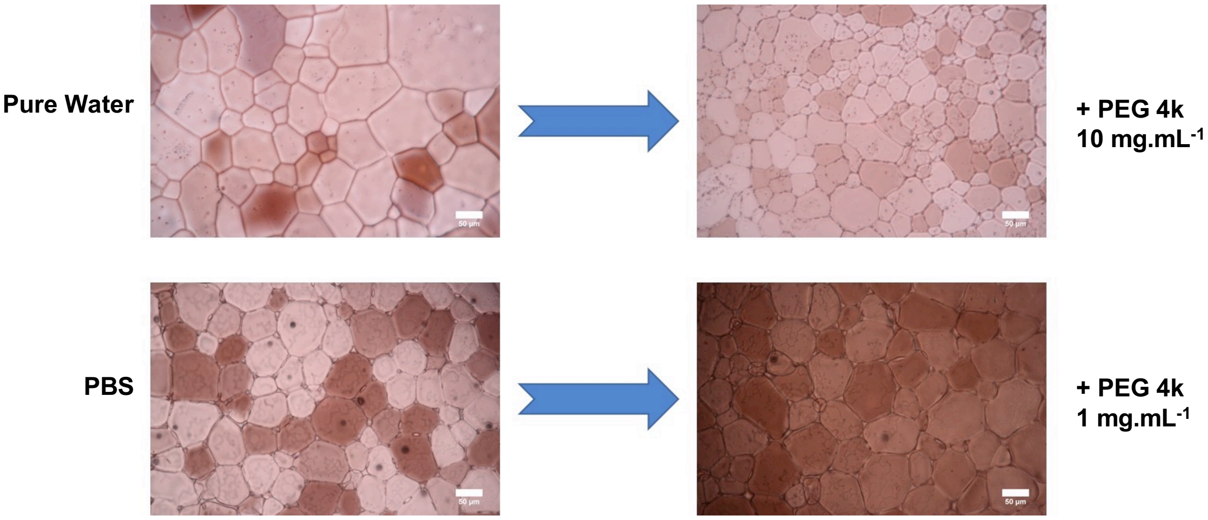

Figure S4. Cryo-micrographs showing ice wafers of grown in either water or PBS alone, and with PEG added in. All micrographs are taken after 30 minutes at – 8 °C. Scale bar = 50 µm.

References

[1] P. L. Davies, B. D. Sykes, *Curr. Opin. Struct. Biol.* **1997**, *7*, 828–834.

[2] P. L. Davies, *Trends Biochem. Sci.* **2014**, *39*, 548–555.

[3] M. M. Harding, P. I. Anderberg, A. D. J. Haymet, *Eur. J. Biochem.* **2003**, *270*, 1381–1392.

[4] I. K. Voets, *Soft Matter* **2017**, *13*, 4808–4823.

[5] W. O. Valarezo, F. T. Lynch, R. J. McGhee, *J. Aircr.* **1993**, *30*, 807–812.

[6] O. Parent, A. Ilinca, *Cold Reg. Sci. Technol.* **2011**, *65*, 88–96.

[7] K. Brockbank, M. Taylor, *Adv. biopreservation* **2007**, *5*, 157–196.

[8] G. John Morris, E. Acton, *Cryobiology* **2013**, *66*, 85–92.

[9] P. Mazur, *Science (80-.).* **1970**, *168*, 939–949.

[10] P. Mazur, in *Life Frozen State* (Eds.: B. Fuller, N. Lane, E.E. Benson), CRC Press,

Boca Raton, **2004**, pp. 3–65.

[11] Y. C. Song, B. S. Khirabadi, F. Lightfoot, K. G. M. Brockbank, M. J. Taylor, *Nat. Biotechnol.* **2000**, *18*, 296–299.

[12] C. Polge, A. U. SMITH, A. S. PARKES, *Nature* **1949**, *164*, 666–666.

[13] B. Wowk, E. Leitl, C. M. Rasch, N. Mesbah-Karimi, S. B. Harris, G. M. Fahy, *Cryobiology* **2000**, *40*, 228–236.

[14] J. F. Carpenter, T. N. Hansen, *Proc. Natl. Acad. Sci.* **1992**, *89*, 8953–8957.

[15] H. Chao, P. L. Davies, J. F. Carpenter, *J. Exp. Biol.* **1996**, *199*, 2071–2076.

[16] S. Matsumoto, M. Matsusita, T. Morita, H. Kamachi, S. Tsukiyama, Y. Furukawa, S. Koshida, Y. Tachibana, S. I. Nishimura, S. Todo, *Cryobiology* **2006**, *52*, 90–98.

[17] M. M. Tomczak, D. K. Hincha, S. D. Estrada, R. E. Feeney, J. H. Crowe, *Biochim. Biophys. Acta - Biomembr.* **2001**, *1511*, 255–263.

[18] B. Rubinsky, A. Arav, M. Mattioli, A. L. Devries, *Biochem. Biophys. Res. Commun.* **1990**, *173*, 1369–1374.

[19] A. Eniade, M. Purushotham, R. N. Ben, J. B. Wang, K. Horwath, *Cell Biochem. Biophys.* **2003**, *38*, 115–124.

[20] D. E. Mitchell, A. E. R. Fayter, R. C. Deller, M. Hasan, J. Gutierrez-Marcos, M. I. Gibson, *Mater. Horizons* **2019**, 10.1039/C8MH00727F.

[21] R. C. Deller, M. Vatish, D. A. Mitchell, M. I. Gibson, *Nat. Commun.* **2014**, *5*, 3244.

[22] C. J. Capicciotti, J. S. Poisson, C. N. Boddy, R. N. Ben, *Cryobiology* **2015**, *70*, 79–89.

[23] J. G. Briard, J. S. Poisson, T. R. Turner, C. J. Capicciotti, J. P. Acker, R. N. Ben, *Sci. Rep.* **2016**, *6*, 23619.

[24] C. I. Biggs, T. L. Bailey, Ben Graham, C. Stubbs, A. Fayter, M. I. Gibson, *Nat. Commun.* **2017**, *8*, 1546.

[25] C. Budke, A. Dreyer, J. Jaeger, K. Gimpel, T. Berkemeier, A. S. Bonin, L. Nagel, C. Plattner, A. L. Devries, N. Sewald, et al., *Cryst. Growth Des.* **2014**, *14*, 4285–4294.

[26] C. A. Knight, D. Wen, R. A. Laursen, *Cryobiology* **1995**, *32*, 23–34.

[27] J. P. Pezacki, M. Noestheden, R. N. Ben, J. Jackman, D. Moffat, S. Findlay, *Biochem. Biophys. Res. Commun.* **2007**, *354*, 340–344.

[28] L. L. C. Olijve, A. S. Oude Vrielink, I. K. Voets, *Cryst. Growth Des.* **2016**, *16*, 4190–4195.

[29] M. M. Tomczak, C. B. Marshall, J. A. Gilbert, P. L. Davies, *Biochem. Biophys. Res. Commun.* **2003**, *311*, 1041–1046.

[30] Z. He, K. Liu, J. Wang, *Acc. Chem. Res.* **2018**, *51*, 1082–1091.

[31] A. T. Rahman, T. Arai, A. Yamauchi, A. Miura, H. Kondo, Y. Ohyama, S. Tsuda, *Sci. Rep.* **2019**, *9*, 2212.

[32] K. Meister, A. L. DeVries, H. J. Bakker, R. Drori, *J. Am. Chem. Soc.* **2018**, *140*, 9365–9368.

[33] K. Mochizuki, V. Molinero, *J. Am. Chem. Soc.* **2018**, *140*, 4803–4811.

[34] A. J. Scotter, C. B. Marshall, L. A. Graham, J. A. Gilbert, C. P. Garnham, P. L. Davies, *Cryobiology* **2006**, *53*, 229–239.

[35] C. B. Marshall, G. L. Fletcher, P. L. Davies, *Nature* **2004**, *429*, 153.

[36] C. Budke, T. Koop, *ChemPhysChem* **2006**, *7*, 2601–2606.

[37] P. M. Naullage, L. Lupi, V. Molinero, *J. Phys. Chem. C* **2017**, *121*, 26949–26957.

[38] T. Congdon, R. Notman, M. I. Gibson, *Biomacromolecules* **2013**, *14*, 1578–1586.

[39] M. I. Gibson, C. A. Barker, S. G. Spain, L. Albertin, N. R. Cameron, *Biomacromolecules* **2009**, *10*, 328–333.

[40] R. C. R. C. R. C. Deller, T. Congdon, M. A. M. A. Sahid, M. Morgan, M. Vatish, D. A. D. A. Mitchell, R. Notman, M. I. M. I. Gibson, *Biomater. Sci.* **2013**, *1*, 478.

[41] N. S. Vail, C. Stubbs, C. I. Biggs, M. I. Gibson, *ACS Macro Lett.* **2017**, *6*, 1001–1004.

[42] T. R. Congdon, R. Notman, M. I. Gibson, *Eur. Polym. J.* **2017**, *88*, 320–327.

[43] R. Drori, C. Li, C. Hu, P. Raiteri, A. L. Rohl, M. D. Ward, B. Kahr, *J. Am. Chem. Soc.*

2016, *138*, 13396–13401.

[44] S. Deville, C. Viazzi, J. Leloup, A. Lasalle, C. Guizard, E. Maire, J. Adrien, L. Gremillard, *PLoS One* **2011**, *6*, e26474.

[45] O. Mizrahy, M. Bar-Dolev, S. Guy, I. Braslavsky, *PLoS One* **2013**, *8*, e59540.

[46] H. Geng, X. Liu, G. Shi, G. Bai, J. Ma, J. Chen, Z. Wu, Y. Song, H. Fang, J. Wang, *Angew. Chemie Int. Ed.* **2017**, *56*, 997–1001.

[47] T. F. Whale, M. Rosillo-Lopez, B. J. Murray, C. G. Salzmann, *J. Phys. Chem. Lett.* **2015**, *6*, 3012–3016.

[48] C. I. Biggs, C. Packer, S. Hindmarsh, M. Walker, N. R. Wilson, J. P. Rourke, M. I. Gibson, *Phys. Chem. Chem. Phys.* **2017**, *19*, 21929–21932.

[49] P. W. Wilson, K. E. Osterday, A. F. Heneghan, A. D. J. Haymet, *J. Biol. Chem.* **2010**, *285*, 34741–34745.

[50] S. Liu, R. N. Ben, *Org. Lett.* **2005**, *7*, 2385–2388.

[51] A. K. Balcerzak, C. J. Capicciotti, J. G. Briard, R. N. Ben, *RSC Adv.* **2014**, *4*, 42682–42696.

[52] C. J. Capicciotti, J. F. Trant, M. Leclère, R. N. Ben, *Bioconjug. Chem.* **2011**, *22*, 605–616.

[53] C. P. Garnham, R. L. Campbell, P. L. Davies, *Proc. Natl. Acad. Sci.* **2011**, *108*, 7363–7367.

[54] M. J. Kuiper, C. J. Morton, S. E. Abraham, A. Gray-Weale, *Elife* **2015**, *4*, e05142.

[55] M. E. Daley, L. Spyracopoulos, Z. Jia, P. L. Davies, B. D. Sykes, *Biochemistry* **2002**, *41*, 5515–5525.

[56] L. L. C. Olijve, K. Meister, A. L. Devries, J. G. Duman, S. Guo, H. J. Bakker, *Proc. Natl. Acad. Sci. USA* **2016**, *113*, 3740–3745.

[57] A. Hudait, D. R. Moberg, Y. Qiu, N. Odendahl, F. Paesani, V. Molinero, *Proc. Natl. Acad. Sci.* **2018**, *115*, 8266–8271.

[58] A. Hudait, N. Odendahl, Y. Qiu, F. Paesani, V. Molinero, *J. Am. Chem. Soc.* **2018**, *140*, 4905–4912.

[59] Y. Qiu, A. Hudait, V. Molinero, *J. Am. Chem. Soc.* **2019**, preprint.

[60] H. Asakawa, G. Sazaki, K. Nagashima, S. Nakatsubo, Y. Furukawa, *Proc. Natl. Acad. Sci.* **2016**, *113*, 1749–1753.

[61] R. Y. Tam, S. S. Ferreira, P. Czechura, R. N. Ben, J. L. Chaytor, *J. Am. Chem. Soc.* **2008**, *130*, 17494–17501.

[62] A. K. Balcerzak, C. J. Capicciotti, J. G. Briard, R. N. Ben, *RSC Adv.* **2014**, *4*, 42682–42696.

[63] J. F. Trant, R. A. Biggs, C. J. Capicciotti, R. N. Ben, *RSC Adv.* **2013**, *3*, 26005–26009.

[64] A. K. Balcerzak, M. Febbraro, R. N. Ben, *RSC Adv.* **2013**, *9*, 3232–3236.

[65] C. J. Capicciotti, M. Leclère, F. A. Perras, D. L. Bryce, H. Paulin, J. Harden, Y. Liu, R. N. Ben, *Chem. Sci.* **2012**, *3*, 1408–1416.

[66] Y. Tachibana, G. L. Fletcher, N. Fujitani, S. Tsuda, K. Monde, S. I. Nishimura, *Angew. Chemie - Int. Ed.* **2004**, *43*, 856–862.

[67] D. E. D. E. Mitchell, M. I. M. I. Gibson, *Biomacromolecules* **2015**, *16*, 3411–3416.

[68] A. Hakim, J. B. Nguyen, K. Basu, D. F. Zhu, D. Thakral, P. L. Davies, F. J. Isaacs, Y. Modis, W. Meng, *J. Biol. Chem.* **2013**, *288*, 12295–12304.

[69] P. M. Naullage, Y. Qiu, V. Molinero, *J. Phys. Chem. Lett.* **2018**, *9*, 1712–1720.

[70] M. I. M. I. Gibson, *Polym. Chem.* **2010**, *1*, 1141.

[71] G. N. Tew, R. W. Scott, M. L. Klein, W. F. Degrado, *Acc. Chem. Res.* **2010**, *43*, 30–9.

[72] M. F. Ilker, K. Nüsslein, G. N. Tew, E. B. Coughlin, *J. Am. Chem. Soc.* **2004**, *126*, 15870–15875.

[73] B. Graham, A. E. R. Fayter, J. E. Houston, R. C. Evans, M. I. Gibson, *J. Am. Chem. Soc.* **2018**, *140*, 5682–5685.

[74] D. E. Mitchell, G. Clarkson, D. J. Fox, R. A. Vipond, P. Scott, M. I. Gibson, *J. Am.*

Chem. Soc. **2017**, *139*, 9835–9838.

[75] K. Matsumura, S.-H. H. Hyon, *Biomaterials* **2009**, *30*, 4842–4849.

[76] D. E. Mitchell, N. R. Cameron, M. I. Gibson, *Chem. Commun.* **2015**, *51*, 12977–12980.

[77] D. E. Mitchell, M. Lilliman, S. G. Spain, M. I. Gibson, *Biomater. Sci.* **2014**, *2*, 1787–1795.

[78] R. Rajan, F. Hayashi, T. Nagashima, K. Matsumura, *Biomacromolecules* **2016**, *17*, 1882–1893.

[79] C. Stubbs, J. Lipecki, M. I. Gibson, *Biomacromolecules* **2017**, *18*, 295–302.

[80] C. Stubbs, T. R. Congdon, M. I. Gibson, *Eur. Polym. J.* **2019**, *110*, 330–336.

[81] R. Rajan, M. Jain, K. Matsumura, *J. Biomater. Sci. Polym. Ed.* **2013**, *24*, 1767–80.

[82] T. Congdon, P. Shaw, M. I. M. I. Gibson, *Polym. Chem.* **2015**, *6*, 4749–4757.
